# Mesenchymal actomyosin contractility is required for androgen-driven urethral masculinization in mice

**DOI:** 10.1038/s42003-019-0336-3

**Published:** 2019-03-08

**Authors:** Alvin R. Acebedo, Kentaro Suzuki, Shinjiro Hino, Mellissa C. Alcantara, Yuki Sato, Hisashi Haga, Ken-ichi Matsumoto, Mitsuyoshi Nakao, Kenji Shimamura, Toru Takeo, Naomi Nakagata, Shinichi Miyagawa, Ryuichi Nishinakamura, Robert S. Adelstein, Gen Yamada

**Affiliations:** 1 Department of Developmental Genetics, Institute of Advanced Medicine, Wakayama Medical University, Wakayama, 641-8509 Japan; 2 Department of Medical Cell Biology, Institute of Molecular Embryology and Genetics (IMEG), Kumamoto University, Kumamoto, 860-0811 Japan; 3 Department of Anatomy and Cell Biology, Graduate School of Medical Sciences, Kyushu University, 3-1-1 Maidashi, Higashi-ku, Fukuoka, 812-8582 Japan; 4 Transdisciplinary Life Science Course, Faculty of Advanced Life Science, Hokkaido University, N10-W8, Kita-ku, Sapporo, 060-0810 Japan; 5 Department of Biosignaling and Radioisotope Experiment, Interdisciplinary Center for Science Research, Organization for Research, Shimane University, Izumo, Shimane, 693-8501 Japan; 6 Department of Brain Morphogenesis, Institute of Molecular Embryology and Genetics (IMEG), Kumamoto University, 2-2-1 Honjo, Chuo-ku, Kumamoto, 860-0811 Japan; 7 Division of Reproductive Engineering, Center for Animal Resources and Development (CARD), Kumamoto University, 2-2-1 Honjo, Chuo-ku, Kumamoto, 860-0811 Japan; 8 Department of Kidney Development, Institute of Molecular Embryology and Genetics (IMEG), Kumamoto University, 2-2-1 Honjo, Chuo-ku, Kumamoto, 860-0811 Japan; 9 Laboratory of Molecular Cardiology, National Heart, Lung, and Blood Institute, National Institutes of Health, Bethesda, MD 20892-1762 USA

## Abstract

The morphogenesis of mammalian embryonic external genitalia (eExG) shows dynamic differences between males and females. In genotypic males, eExG are masculinized in response to androgen signaling. Disruption of this process can give rise to multiple male reproductive organ defects. Currently, mechanisms of androgen-driven sexually dimorphic organogenesis are still unclear. We show here that mesenchymal-derived actomyosin contractility, by MYH10, is essential for the masculinization of mouse eExG. MYH10 is expressed prominently in the bilateral mesenchyme of male eExG. Androgen induces MYH10 protein expression and actomyosin contractility in the bilateral mesenchyme. Inhibition of actomyosin contractility through blebbistatin treatment and mesenchymal genetic deletion induced defective urethral masculinization with reduced mesenchymal condensation. We also suggest that actomyosin contractility regulates androgen-dependent mesenchymal directional cell migration to form the condensation in the bilateral mesenchyme leading to changes in urethral plate shape to accomplish urethral masculinization. Thus, mesenchymal-derived actomyosin contractility is indispensable for androgen-driven urethral masculinization.

## Abstract (web summary)

Alvin Acebedo, Kentaro Suzuki et al. show that mesenchymal-derived actomyosin contractility is required for androgen-dependent urethral masculinization in mouse embryos. They also show that actomyosin contractility regulates mesenchymal cell migration during the developmental process.

## Introduction

Androgens are steroid hormones that are essential for the masculinization of the male reproductive tract such as the external genitalia, epididymis, and other organs^1,2^. Defects of androgen signaling lead to various developmental defects in male-type sexually characteristic organogenesis. However, the molecular mechanisms underlying androgen signaling and how these regulate sexually dimorphic organogenesis are still unclear.

The development of mammalian external genitalia gives rise to sexually dimorphic structures, the male and female external genitalia^3,4^. Embryonic external genitalia (eExG, also known as genital tubercle) develop as an outgrowing organ^4,5^. During eExG outgrowth, the urethral plate epithelium (UPE) forms continuously from the proximal to the distal region (glans) of the eExG within the midline region. During development of the eExG in response to androgen signaling, the UPE forms a male-specific tubular urethra (hereafter designated as urethral masculinization)^3,6^. Disruption in androgen signaling gives rise to congenital anomalies including defects of urethral formation or hypospadias. Hypospadias encompasses phenotypes involving ventral ectopic urethral openings^7^. The etiology of defects of urethral formation is likely to be multifaceted, involving multiple genetic anomalies (Supplementary Table 1). Thus, urethral masculinization in the mouse embryonic external genitalia provides a unique model to study the mechanisms of androgen-induced sexually dimorphic organ development.

Previously, we identified that androgen signaling in the mesenchyme adjacent to the UPE (hereafter designated as bilateral mesenchyme) is required for urethral masculinization^6^. Several sexually dimorphic genes such as *Mafb* (v-maf avian musculoaponeurotic fibrosarcoma oncogene homolog B)^6^, *β-catenin*^3,8^, and *Srd5α2* (5α-reductase type 2 mRNA)^9^ are expressed in male bilateral mesenchyme under androgen signaling. Furthermore, knockout mice for *Mafb* and *β-catenin* in the mesenchyme show urethral developmental defects^3,6^. Additionally, it was reported that mesenchymal F-actin shows sexually dimorphic expression pattern in the eExG bilateral mesenchyme^10^.

Actomyosin is a cytoskeletal system composed of an F-actin network bound to the motor protein nonmuscle myosin II. Nonmuscle myosin heavy chains, which are essential components of nonmuscle myosin II, exist in three isoforms (NMHCIIA, NMHCIIB, and NMHCIIC) with each isoform encoded by the *Myh9*, *Myh10*, and *Myh14* genes respectively^11^. Mechanical force is generated through the ATP-dependent contraction of nonmuscle myosin II which is then transmitted to the F-actin network and through cell–cell and cell-ECM interactions. Actomyosin contractility plays significant roles in various cellular processes such as cell adhesion and cell migration^12^. Regulation of these cell processes by actomyosin is a driving mechanism in tissue morphogenesis. Actomyosin contractility also regulates changes in epithelial cell shape, which facilitates organogenesis. In the development of the neural tube, epithelia-derived actomyosin contractility induces apical constrictions, which results in the folding of the neural plate epithelium^13^. Furthermore, epithelial actomyosin contractility by *Myh9* and *Myh10* elicits tissue fusion during organogenesis^11,14,15^. Thus, the involvement of actomyosin contractility in shaping organogenesis has long been reported but is usually highlighted in the epithelia rather than mesenchyme. Currently, the contribution of actomyosin contractility in mesenchyme during organogenesis is not well understood.

In this study, we investigated the role of actomyosin during urethral masculinization. Intriguingly, *Myh10* was expressed prominently and actomyosin contractility was increased in the condensed mesenchyme of the male eExG in an androgen-dependent manner during urethral masculinization. To analyze mesenchymal-derived actomyosin contractility, we established a slice culture system to recapitulate mouse embryonic urethral formation in vitro. Both mouse genetic study and chemical inhibitor experiments using the slice culture system suggest that actomyosin contractility is required for androgen-driven urethral masculinization. We thus report here a unique role for mesenchymal-derived actomyosin contractility for sexually dimorphic organogenesis.

## Results

### Dynamic urethral masculinization processes of mouse eExG

**Fig. 1:**
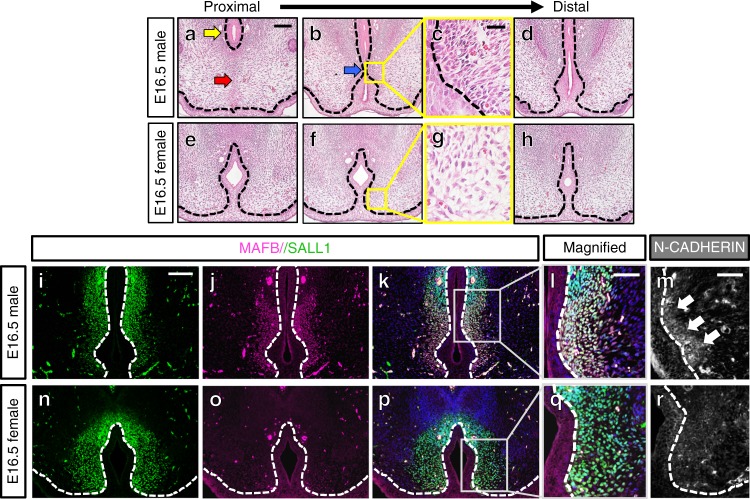
Masculinization processes of the mouse urethra in E16.5 eExG. **a**–**h** H&E staining of E16.5 eExG. **a** Proximal male eExG showing tubular urethra (yellow arrow) and confluent bilateral mesenchyme (red arrow) after removal of the UPE from the midline. **b** Medial curvature of the UPE prior to the removal from the midline (blue arrow). **c** Magnified view of male eExG condensed bilateral mesenchyme. **d** Distal male eExG. **e** Proximal female eExg. **f** Middle female eExG. **g** Magnified view of female eExG uncondensed bilateral mesenchyme. **h** Distal female eExG. UPE is retained in all regions of the female eExG without medial curvature at E16.5. **i**–**r** Expression of bilateral mesenchymal markers SALL1, MAFB, and N-CADHERIN in both male and female eExG. SALL1 is expressed in both male and female bilateral mesenchyme while MAFB and N-CADHERIN show sexually dimorphic expression. **i** SALL1 expression in the male eExG. **j** MAFB expression in the male eExG. **k** Merged SALL1 and MAFB expression in male eExG. **l** Magnified view of male bilateral mesenchyme showing overlapping SALL1 and MAFB expression. **m** N-CADHERIN expression in the male bilateral mesenchyme (white arrows). **n** SALL1 expression in the female bilateral mesenchyme. **o** Lack of MAFB expression in the female bilateral mesenchyme. **p** Merged SALL1 and MAFB expression in female eExG. **q** Magnified view of female bilateral mesenchyme showing SALL1 expression. **r** reduced N-CADHERIN expression in the female bilateral mesenchyme. Dashed lines; epithelial-mesenchymal border. Squares in **b**, **f**, **k**, **p**; representative areas of the SALL1-expressing bilateral mesenchyme. Scale bars in **a**, **i** = 100 µm, scale bar in **c** = 20 µm, scale bars in **l**, **m** = 50 µm

During urethral masculinization of male eExG at E16.5, the UPE showed morphological changes characteristic of tissue fusion (Fig. 1), such as removal of midline epithelial structures and mesenchymal confluence^16^. Formation of a male-specific tubular urethra (urethral masculinization) occurs proximally to distally during eExG development (Fig. 1a–d). The UPE curved medially and became thinner at the fusion site (Fig. 1b; blue arrow), eventually disappearing from the midline of the eExG (Fig. 1a). Disappearance of the UPE at the fusion site led to the confluence of the bilateral mesenchyme at the mid-ventral region of the eExG. Remaining UPE subsequently developed into the tubular urethra (Fig. 1a; yellow arrow). Confluent bilateral mesenchyme ventral to the tubular urethra differentiated simultaneously into a mesenchymal seam (Fig. 1a; red arrow). In female eExg, the UPE did not curve medially throughout its development (Fig. 1e–h).

SALL1 was found to be broadly expressed in both male and female mesenchyme adjacent to the urethral plate (Fig. 1i, n). On the other hand, MAFB, which is an essential masculinization gene for urethra^6^, was strongly expressed in the male bilateral mesenchyme but not in the female mesenchyme (Fig. 1j, o). Expression of SALL1 overlapped with MAFB in the bilateral mesenchyme (Fig. 1k, l). Thus, we defined SALL1 as a marker indicating the bilateral mesenchyme in both male and female eExG. Intriguingly, the SALL1-expressing bilateral mesenchyme in male eExG condensed prominently at the fusion site during urethral masculinization (Fig. 1b; square, c, l). On the other hand, female eExG retained the uncondensed bilateral mesenchyme (Fig. 1f; square, g, q). We subsequently investigated the expression of the cell-cell adhesion marker, N-CADHERIN. N-CADHERIN has been previously reported to be necessary for cell condensations^17,18^. N-CADHERIN was expressed prominently in male mesenchymal condensed region but not in female mesenchyme (Fig. 1m, r). Thus, the bilateral mesenchyme condenses close to the fusing UPE, which suggests its role for urethral masculinization.

### Sexually dimorphic MYH10 expression in eExG mesenchyme

**Fig. 2:**
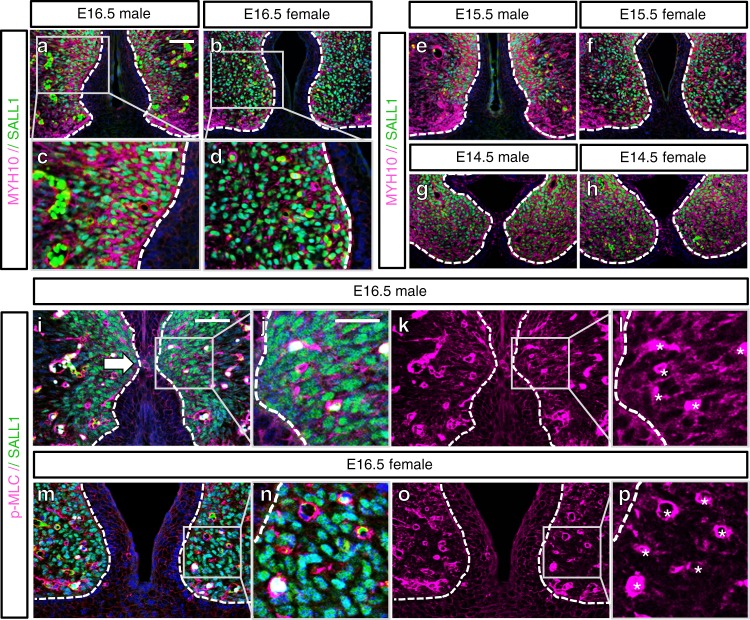
Sexually dimorphic expression of actomyosin cytoskeletal components in eExG bilateral mesenchyme. **a**–**h** MYH10 expression in the SALL1-expressing bilateral mesenchyme of **a** E16.5 male eExG and **b** E16.5 female eExG with higher male expression. Magnified view of SALL1-expressing mesenchyme in **c** male eExG and **d** female eExG. MYH10 expression in **e** E15.5 male eExG, **f** E15.5 female eExG, **g** E14.5 male eExG, **h** E14.5 female eExG. MYH10 show increased expression in male eExG starting at E15.5. **i**–**p** Confocal images showing p-MLC expression in the SALL1-expressing mesenchyme in **i**–**l** E16.5 male eExG and **m**–**p** E16.5 female eExG. **j**, **l** Magnified views of male SALL1-expressing male bilateral mesenchyme showing higher expression of p-MLC compared to **n**, **p** female bilateral mesenchyme. Dashed lines; epithelial–mesenchymal border. Squares in **a**, **b**, **i**, **m**, **k**, **o**; representative areas of the SALL1-expressing bilateral mesenchyme. White arrow in **i**; epithelial fusion site. Asterisks in **l**, **p**; fluorescent signal from non-bilateral mesenchyme (vasculature). Scale bars in **a**, **i** = 50 µm, scale bars in **c**, **j** = 25 µm

Previously, we reported that androgen regulates sexually dimorphic F-actin assemblies in the genital organogenesis^10^. Thus, we investigated further on actomyosin during urethral masculinization. One of the components of actomyosin, MYH10, was expressed prominently in the male SALL1-expressing bilateral mesenchyme at E16.5 while female eExG showed lower expression (Fig. 2a–d). In contrast, MYH9 was expressed mainly in the UPE and ectodermal epithelia without showing sexual dimorphism in E16.5 eExG. MYH9 also showed weak expression in the bilateral mesenchyme (Supplementary Fig. 1). Phosphorylation of the myosin light chain (p-MLC) is essential for the increased contractility of actomyosin indicating its role in force generation^11,12^. Male eExG showed higher p-MLC expression in the bilateral mesenchyme and urethral epithelia near the fusion site (Fig. 2i–l) compared to female eExG at E16.5 (Fig. 2m–p). We further investigated the onset of MYH10 sexually dimorphic expression during urethral masculinization. The MYH10 expression pattern appeared similar between male and female eExG mesenchyme at E14.5 (Fig. 2g, h). MYH10 expression became higher in male eExG mesenchyme starting at E15.5 when urethral masculinization can be first observed (Fig. 2e, f). These results suggest a correlation between the spatiotemporal expression of MYH10 and the occurrence of sexually dimorphic development of the eExG.

### Establishment of a eExG slice culture system

**Fig. 3:**
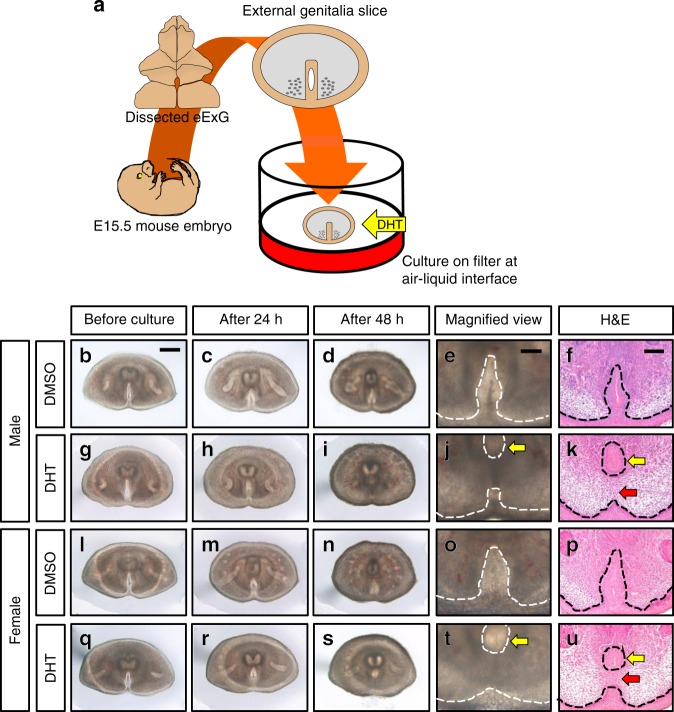
eExG slices show androgen-driven urethral masculinization after 48 h culture. **a** Illustration of the methodology for eExG slice culture. **b**–**k** Male eExG slices treated with **b**–**f** DMSO (*n* = 15 eExG slices; Table 1) or with **g**–**k** DHT (*n* = 39 eExG slices; Table 1) **b**, **g** before culture, **c**, **h** after 24 h culture and **d**, **e**, **i**, **j** after 48 h culture. **e**, **j** Magnified view of male eExG slices after 48 h culture. **f**, **k** H&E staining of male eExG slices after 48 h culture. **l**–**u** Female eExG slices treated with **l**–**p** DMSO (*n* = 32 eExG slices; Table 1) or with **q**–**u** DHT (*n* = 30 eExG slices; Table 1) **l**, **q** before culture, **m**, **r** after 24 h and **n**, **o**, **s**, **t** after 48 h culture. **o**, **t** Magnified view of female eExG slices after 48 h culture. **p**, **u** H&E staining of female eExG slices after 48 h culture. Dashed lines; epithelial-mesenchymal border. Yellow arrows in **j**, **k t**, **u**; tubular urethra. Red arrows in **k**, **u**; mesenchymal seam. Scale bar in **b** = 300 µm, scale bars in **e**, **f** = 100 µm

To analyze the mechanism of urethral masculinization efficiently, we established a new eExG slice culture system (Fig. 3a, see Methods for details). eExG slices showed urethral tube-like structure in both male and female slices after 48 h with 10^−8^ M 5α-Dihydrotestosterone (DHT), which is a major androgen for the masculinization of external genitalia (Fig. 3i, j, s, t; yellow arrow). Further histological analyses clearly showed the urethral tube structure (Fig. 3k, u; yellow arrow) and mesenchymal seam (Fig. 3k, u; red arrow) in DHT treated slices. In contrast, no tube-like structures were observed in both male and female eExG slices cultured without DHT (Fig. 3d–f, n–p).

Each specimen was scored by five stages (Score 0 to Score 4) according to histological observations by hematoxylin and eosin (H&E) staining and whole mount images of eExG slices (see Methods for details, Supplementary Fig. 2). Such quantification of fusion revealed that 43.6% (a total of 39 eExG slices) of DHT-treated male eExG slices developed a tubular urethra and a mesenchymal seam (Score 3 and 4). On the other hand, DMSO-treated male eExG slices showed no visible tubularization. Similar to male eExG slices, 33.3% (a total of 30 eExG slices) of DHT-treated female eExG slices developed a tubular urethra and mesenchymal seam in contrast to DMSO-treated female eExG slices (Table 1, Supplementary Data, Fig. 3, Supplementary Fig. 2). A significant association between the presence of DHT during culture and urethral masculinization was observed for both male (*n* = 54, *p* = 0.002) and female eExG slices (*n* = 62, *p* = 0.0019). Confocal live imaging analyses further confirmed that male eExG slices cultured with DHT underwent masculinization with the formation of tubular urethra from the UPE (Supplementary Movie 1). All DHT-treated eExG slices showed increased condensation of the bilateral mesenchyme compared to DMSO-treated eExG slices. Additionally, DHT-treated eExG slices showed induced expression of the androgen-responsive genes, AR, MAFB and β-CATENIN (Supplementary Fig. 3). E-CADHERIN was expressed in eExG urethral and ectodermal epithelia suggesting the retention of the epithelial structural integrity (Supplementary Fig. 4). These results indicate that the current eExG slice culture system recapitulates the androgen-driven embryonic urethral masculinization. Thus, we used this system to further analyze the contribution of the eExG mesenchyme to urethral masculinization.

**Table 1:** Incidence of urethral masculinization in eExG slice cultures in response to DHT treatment

	Male		Female	
	DMSO	DHT	DMSO	DHT
Score 4	0	12	0	6
Score 3	0	5	1	4
Score 2	3	6	4	1
Score 1	5	3	20	5
Score 0	7	13	7	14
TOTAL	15	39	32	30

### Blebbistatin inhibits androgen-driven urethral masculinization

**Fig. 4:**
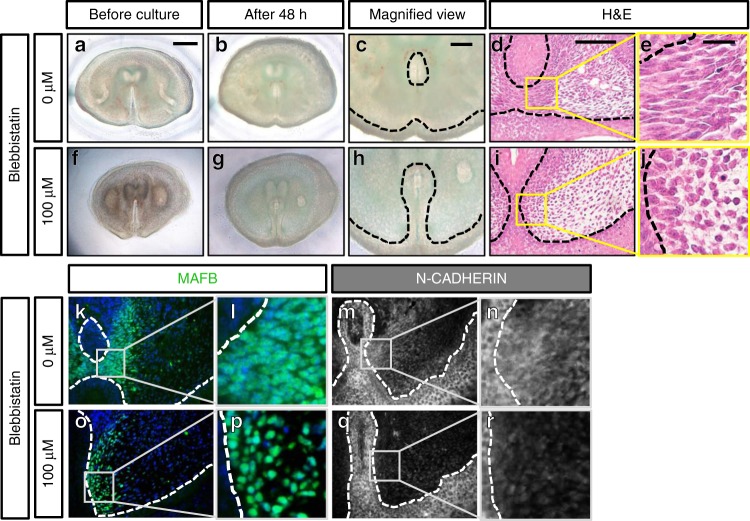
Defective androgen-driven urethral masculinization by blebbistatin treatment. **a**–**e** Control male eExG slice (*n* = 18 eExG slices; Table 2) and **f**–**j** blebbistatin-treated male eExG slice (*n* = 21 slices; Table 2) **a**, **f** before culture and **b**, **c**, **g**, **h** after 48 h culture with DHT. **c**, **h** Magnified view showing urethral morphology of **c** control male eExG slice and **h** blebbistatin-treated male eExG slice at 48 h. **d**, **e**, **i**, **j** H&E staining and **k**, **l**, **o**, **p** MAFB immunostaining of **d**, **e**, **k**, **l** control male eExG slice (*n* = 4 eExG slices) and **i**, **j**, **o**, **p** blebbistatin-treated male eExG slice (*n* = 4 eExG slices) after 48 h culture showing defects in mesenchymal condensation. **m**, **n**, **q**, **r** N-CADHERIN expression in **m**, **n** control male eExG slice (*n* = 3 eExG slices) and **q**, **r** blebbistatin-treated male eExG slice (*n* = 3 eExG slices) after 48 h culture. Dashed lines; epithelial-mesenchymal border. Squares in **d**, **i**, **k**, **o**, **m**, **q**; magnified view of bilateral mesenchyme. Scale bars in **a** = 300 µm. Scale bars in **c**, **d** = 100 µm. Scale bar in **e** **=** 20 µm

We further analyzed the functional relevance of actomyosin contractility in androgen-driven urethral masculinization using male eExG slices treated with the nonmuscle myosin II inhibitor, blebbistatin (Fig. 4; Table 2, Supplementary Data). In control male eExG slices treated with DHT alone, 55.6% of treated eExG slices were assigned as Score 3 or 4 (a total of 18 eExG slices). In contrast, only 19.1% of male eExG slices treated with both blebbistatin and DHT were assigned as Score 3 or 4 after 48 h culture (a total of 21 eExG slices). Blebbistatin treatment resulted in a greater incidence of retention of the UPE in eExG slices (Fig. 4g, h). Score 1 was assigned to 52.4% of blebbistatin-treated male eExG slices (11 out a total of 21 eExG slices). In comparison, only 16.7% of control eExG slices were assigned as Score 1 (3 out a total of 18 eExG slices; Table 2). Furthermore, there was a significant association between blebbistatin treatment and inhibition of urethral masculinization (*n* = 39, *p* = 0.0178). These results suggest that actomyosin contractility is necessary for androgen-driven urethral masculinization. We further analyzed the effect of blebbistatin on mesenchymal condensation. Histological analysis using MAFB revealed that control eExG slices developed prominent mesenchymal condensations (Fig. 4d, e, k, l). In contrast, blebbistatin treatment reduced mesenchymal condensation (Fig. 4i, j, o, p). Actomyosin contractility thus contributes to formation of mesenchymal condensations.

**Table 2:** Incidence of urethral masculinization inhibition in response to blebbistatin treatment

	Blebbistatin	
	0 µM (+DHT)	100 µM (+DHT)
Score 4	9	1
Score 3	1	3
Score 2	0	5
Score 1	3	11
Score 0	5	1
TOTAL	18	21

N-CADHERIN was expressed in MYH10-expressing cells indicating the possibility of actomyosin-mediated regulation of cell–cell adhesion in the bilateral mesenchyme (Supplementary Fig. 5). The condensed mesenchyme of control eExG slices is defined by increased N-CADHERIN expression (Fig. 4m, n). Consistent with the current analysis, blebbistatin-treated eExG slices showed prominent reduction in mesenchymal N-CADHERIN expression indicating disruption of cell–cell adhesions in response to actomyosin inhibition (Fig. 4q, r). These results suggest that actomyosin contractility regulates cell–cell adhesions in the bilateral mesenchyme during urethral masculinization.

Mesenchymal condensation forms in association with directed migration of cells resulting in locally increased cell density^17,19^. In eExG, male mesenchymal cells migrate toward the UPE during embryonic urethral masculinization in an androgen dependent manner^10^. To directly observe the effects of blebbistatin to androgen-driven mesenchymal cell migration, we performed confocal live imaging of E15.5 male eExG slices. Live imaging analyses revealed that mesenchymal cells migrated toward the UPE in the control male eExG slice (Supplementary Movie 2), while blebbistatin treatment inhibited such cellular behaviors (Supplementary Movie 3). Tracking of individual cells showed clearly that inhibition of actomyosin reduced the directional movement of mesenchymal cell towards the UPE (Supplementary Movie 4, 5). These results suggest that actomyosin regulates androgen-driven mesenchymal cell migration during urethral masculinization.

### Mesenchymal *Myh9/10* is required for urethral masculinization

**Fig. 5:**
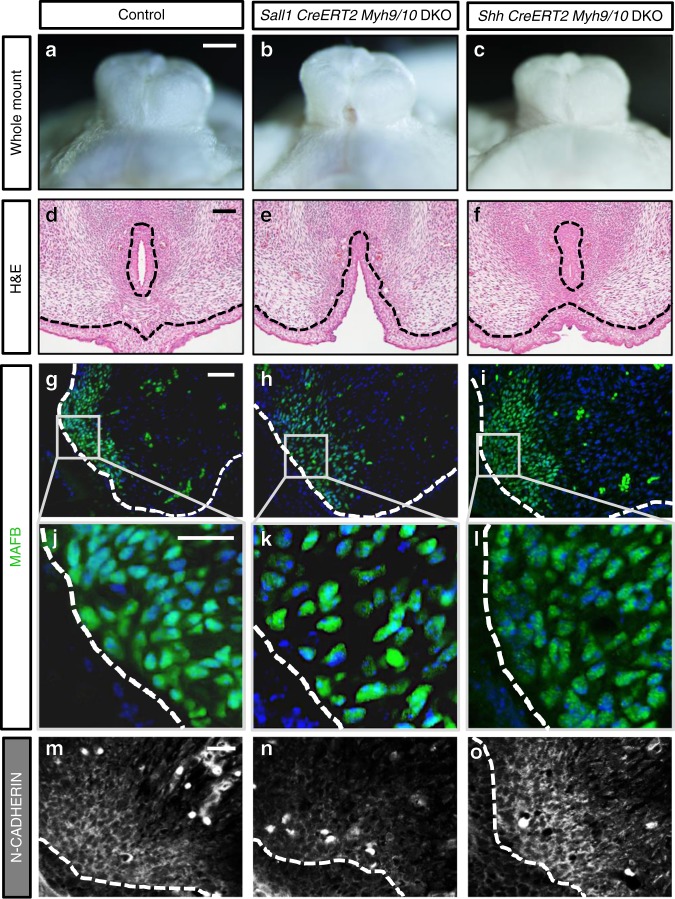
Mesenchymal deletion of *Myh9* and *Myh10* induces defective urethral masculinization with unfused ventral cleft in the male eExG. **a**–**c** Whole images of E18.5 male eExG. **a** Control eExG (*Myh9*^*lox/lox*^*; Myh10*^*lox/lox*^), **b** *Sall1 CreERT2 Myh9/10* DKO eExG and **c** *Shh CreERT2 Myh9/10* DKO eEXG. **d**–**f** H&E staining of E16.5 male eExG. **d** Control eExG, **e** *Sall1 CreERT2 Myh9/10* DKO eExG (*n* = 31 eExG) and **f** *Shh CreERT2 Myh9/10* DKO eExG (*n* = 15 eExG). **g**–**l** MAFB immunostaining of E16.5 male eExG showing mesenchymal condensation in **g**, **j** control eExG (*n* = 3 eExG), **h**, **k** *Sall1 CreERT2 Myh9/10* DKO (*n* = 3 eExG) and **i**, **l** *Shh CreERT2 Myh9/10* DKO eExG (*n* = 3 eExG). **m**–**o** N-CADHERIN expression in **m** control eExG (*n* = 4 eExG), **n** *Sall1 CreERT2 Myh9/10* DKO eExG (*n* = 4 eExG) and **o** *Shh CreERT2 Myh9/10* DKO eExG (*n* = 3 eExG). Tamoxifen (200 mg/kg body weight) was administered to pregnant mice at E9.5. Dashed lines; epithelial-mesenchymal border. Squares in **g**–**i**; magnified view of bilateral mesenchyme. Scale bar in **a** = 500 µm, scale bar in **d** = 100 µm, scale bar in **g** = 50 µm, scale bars in **j**, **m** = 20 µm

We examined the role of mesenchymal actomyosin contractility by utilizing the *Sall1*^*CreERT2/+*^ driver mice (*Sall1 CreERT2*) to delete MYH10 expression in the eExG bilateral mesenchyme (Supplementary Fig. 6a). *Sall1*^*CreERT2/+*^*; Myh10*^*lox/lox*^ knockout mice (hereafter designated as *Sall1 CreERT2 Myh10*) did not show severe defects in urethral masculinization indicating functional compensation by MYH9 (Supplementary Fig. 7). These results prompted us to investigate *Sall1*^*CreERT2/+*^*; Myh9*^*lox/lox*^*; Myh10*^*lox/lox*^ double knockout mice (hereafter designated as *Sall1 CreERT2 Myh9/10* DKO). *Sall1 CreERT2 Myh9/10* DKO mice showed defective urethral masculinization with unfused ventral cleft (Fig. 5b, e). Additionally, the UPE did not curve medially in the *Sall1 CreERT2 Myh9/10* DKO mice (Fig. 5e, compared with Fig. 1b). In these mice, the mesenchyme also showed reduced condensation and N-CADHERIN expression, similar to the blebbistatin-treated eExG slices (Fig. 5h, k, n). Since the *Sall1 CreERT2* driver mice showed mosaic *Cre* recombinase expression in the urethral plate (Supplementary Fig. 6a), we investigated the phenotype of knockout mice using the *Shh CreERT2* driver mice. In these mutant mice, *Cre* recombinase was expressed specifically in the UPE (Supplementary Fig. 6b). *Shh*^*CreERT2/+*^*; Myh9*^*lox/lox*^*; Myh10*^*lox/lox*^ double knockout mice (hereafter designated as *Shh CreERT2 Myh9/10* DKO) did not show obvious defects in urethral masculinization (Fig. 5c, f) and mesenchymal condensation (Fig. 5i, l). Moreover, their mesenchymal N-CADHERIN expression was unaffected by *Myh9* and *Myh10* deletion in the UPE (Fig. 5o). These results strongly suggest that mesenchymal-derived rather than epithelia-derived actomyosin contractility regulates urethral masculinization.

**Fig. 6:**
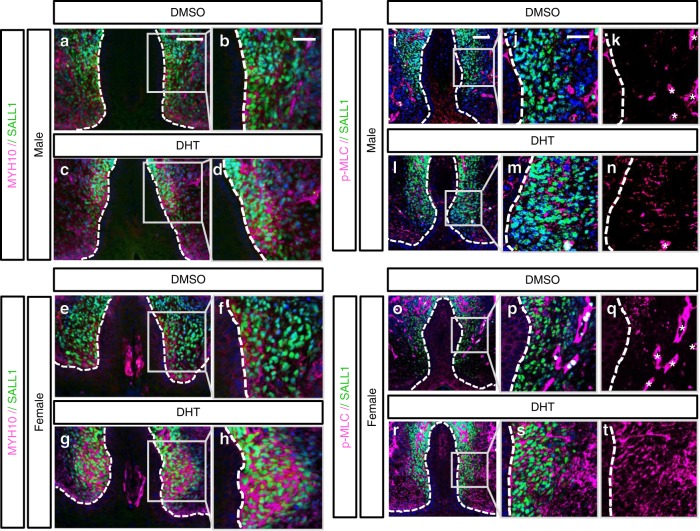
Androgen responsiveness of actomyosin in the bilateral mesenchyme of male and female eExG slices. **a**–**h** MYH10 expression in SALL1-expressing bilateral mesenchyme of **a**, **b** DMSO-treated male eExG slice (*n* = 8 eExG slices), **c**, **d** DHT-treated male eExG slice (*n* = 8 eExG slices), **e**, **f** DMSO-treated female eExG slice (*n* = 10 eExG slices) and **g**, **h** DHT-treated female eExG slice (*n* = 7 eExG slices) after 48 h culture. Magnified view of representative parts of the SALL1-expressing bilateral mesenchyme showing increased MYH10 expression after DHT treatment in both **d** male and **h** female eExG slices. **i**–**t** Confocal images showing p-MLC expression in SALL1-expressing bilateral mesenchyme of **i**–**k** DMSO-treated male eExG slice (*n* = 3 eExG slices), **l**–**n** DHT-treated male eExG slice (*n* = 3 eExG slices), **o**–**q** DMSO-treated female eExG slice (*n* = 4 eExG slices) and **r**–**t** DHT-treated female eExG slice (*n* = 4 eExG slices) after 48 h culture. Magnified view of representative parts of the SALL1-expressing bilateral mesenchyme showing increased p-MLC expression after DHT treatment in both **m**, **n** male and **s**, **t** female eExG slices. Dashed lines; epithelial–mesenchymal border. Squares; representative areas of the SALL1-expressing bilateral mesenchyme. Asterisks in **k**, **n**, **q**; fluorescent signal from non-bilateral mesenchyme (vasculature). Scale bars in **a**, **i** = 50 µm, scale bars in **b**, **j** = 25 µm

Finally, we investigated whether mesenchymal actomyosin contractility is dependent on androgen signaling, we cultured eExG slices in the presence or absence of DHT. DHT-treated male eExG slices displayed increased expression of MYH10 (Fig. 6c, d) and p-MLC (Fig. 6l–n). In contrast, DMSO-treated male eExG slices showed lower MYH10 (Fig. 6a, b) and p-MLC expression (Fig. 6i–k) after 48 h of culture. The expression of MYH10 (Fig. 6e, f) and p-MLC (Fig. 6o–q) was low in the female bilateral mesenchyme. These expressions were induced in the mesenchyme of female eExG slices after DHT treatment (Fig. 6g, h, r–t). These results suggest that mesenchymal-derived actomyosin contractility in the eExG is androgen-dependent.

## Discussion

**Fig. 7:**
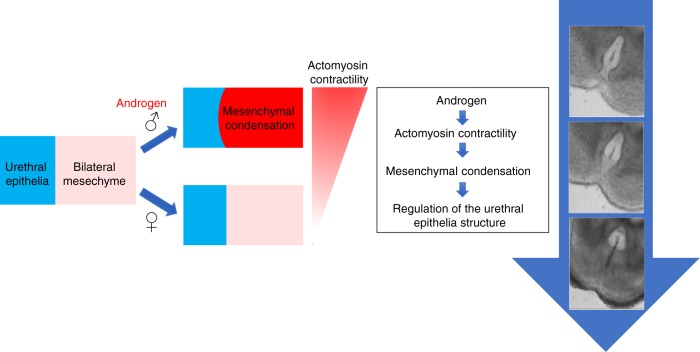
Androgen induces actomyosin contractility, which condenses eExG bilateral mesenchyme and regulates morphogenesis of the urethral epithelia structure during urethral masculinization

Actomyosin contractility is a highly conserved mechanism underlying cell adhesion, cell migration, cell shape and tissue morphogenesis^12,20,21^. Regulation of cell adhesion and cell migration contributes to different processes of organogenesis^16,22^. Previous reports on the role of actomyosin contractility during embryogenesis focused on the contribution of epithelial nonmuscle myosin II^13^. In palatal tissue fusion, epithelia-derived actomyosin contractility regulates the removal of medial epithelial structures, which leads to subsequent confluence of the palatal mesenchyme^23^. In this study, we report a mechanism wherein mesenchymal-derived actomyosin contractility can also contribute to the removal of the midline epithelia during tissue fusion in the urethral masculinization of the eExG. Disruption in androgen signaling leads to congenital anomalies including hypospadias which encompasses phenotypes involving ventral ectopic urethral openings^7^. Previously, we showed that mesenchymal androgen signaling is essential for urethral masculinization by using *Sall1 CreERT2* driver mice to ablate androgen receptor function^6^. In the *Sall1 CreERT2* driver mouse model, *Cre* recombinase is expressed in the bilateral mesenchyme. MYH10 and p-MLC were expressed prominently in the male bilateral mesenchyme in an androgen dependent manner. Furthermore, *Sall1 CreERT2 Myh9/10* DKO mice showed defects in urethral masculinization. These results indicate that androgen-induced, mesenchymal-derived actomyosin contractility is essential for urethral masculinization. Actomyosin contractility thus represents a process for the regulation of sexually dimorphic organogenesis (Fig. 7).

We identified that the urethral masculinization of the eExG was characterized by the androgen-driven condensation of the bilateral mesenchyme. Mesenchymal condensations have been observed during the development of a wide range of organs such as tooth, feather buds, gut villi, ear, and limb^17,19,24–26^. Mechanical dynamics arising from actomyosin-regulated mesenchymal condensations have been implicated in changes in the architectures of adjacent epithelia leading to tissue folding. Different patterns of mesenchymal condensations can induce various types of tissue folding^25^. Mathematical modeling for mesenchymal-epithelial interactions also reveals that mesenchymal-generated mechanical force is sufficient to induce changes in epithelial shape^27^. The mesenchymal condensation in the male eExG may exert force on the UPE leading to initial medial curvature and eventual removal of the UPE. In the current study, loss of mesenchymal condensation due to actomyosin inhibition resulted in reduced epithelial curvature leading to defects of urethral masculinization. Proper regulation of cell proliferation is generally required for organogenesis. Androgen regulates negatively cell proliferation in bilateral mesenchyme during urethral masculinization^9^. This androgen-mediated regulation of cell proliferation and other cell behaviors such as migration and condensation are likely to play roles during urethral masculinization. However, blebbistatin treatment did not cause prominent differences in cell proliferation (Supplementary Fig. 8). Our results strongly suggest that cell migration and the formation of mesenchymal condensation by androgen-induced actomyosin contractility is a sexually dimorphic process to regulate morphogenesis of the urethral epithelia during urethral masculinization. Previous studies have reported that androgen can regulate myosin heavy chain expression in muscle tissue such as cardiomyocytes^28–30^. However, this study is the first report, to our knowledge, to show the sexually dimorphic expression pattern of MYH10 and p-MLC during organogenesis. Currently, it is unclear how androgen regulates actomyosin contractility. One possible mechanism is through the RhoA (Ras homolog gene family, member A)-ROCK (Rho-associated protein kinase) pathway which is a major regulatory mechanism for actomyosin contractility^31^. It has been reported that RhoA is a mediator of androgen-regulated migration in prostate cancer cells^32^. Further investigation on the regulatory mechanisms of androgen-induced actomyosin contractility during urethral masculinization is necessary.

Another possible role of mesenchymal condensation is the regulation of the expression of masculinization genes. Cell condensations are known to lead to changes in gene expression during organogenesis^19,33^. Previously, it was reported that mechanical forces could induce nuclear translocation of β-CATENIN^24,34^. In the eExG, β-CATENIN is a known masculinizing factor induced by androgen signaling. *β-catenin* conditional knockout mice results in defects of urethral formation^3^. The contribution of actomyosin-regulated mesenchymal condensation for the gene expression of masculinizing factors such as β-CATENIN may be important for urethral masculinization. Further analysis on the regulation of mechanosensitive pathways downstream to androgen-driven actomyosin contractility will thus deepen current understanding on sexually dimorphic development.

The condensation of mesenchyme is defined by cell–cell adhesions through increased N-CADHERIN expression^18^. Both pharmacologic inhibition of actomyosin contractility and genetic deletion of *Myh9* and *Myh10* resulted in reduced N-CADHERIN expression. In a previous study, it was reported that nonmuscle myosin II activity regulates the accumulation of cadherins at cell–cell adhesion sites^35^. Furthermore, mouse embryos with ablated *Myh9* develop defects in cell–cell adhesions resulting in disruption of tissue morphology^36^. Similarly, *Myh9* and *Myh10* deletion also impairs cell–cell adhesions with reduced N-CADHERIN expression in kidney metanephric mesenchyme^37^. Our results indicate the requirement of actomyosin contractility for cell–cell adhesion in male eExG bilateral mesenchyme during androgen-driven urethral masculinization. Mesenchymal condensations are also associated with directed cell migration, which results in increased cell density^17^. Cell migration is regulated by actomyosin contractility which is transmitted throughout the cell by F-actin^12^. In the eExG, male mesenchyme shows a unique pattern of F-actin consistent with the directional cell migration toward the UPE. This directional cell migration is responsive to androgen signaling and may be crucial for urethral morphogenesis during masculinization^10^. In this study, we showed that androgen induced actomyosin contractility regulating the male-type directional cell migration. Inhibition of actomyosin contractility drastically reduced androgen-induced directional cell migration and prevented urethral masculinization. Thus, it is assumed actomyosin-regulated directed cell migration contributes to condensation of the bilateral mesenchyme and to urethral masculinization.

In this study, we established a new slice culture system to further analyze efficiently urethral masculinization. Several different organ culture systems have been established to study organogenesis^23,38,39^. However, previous eExG organ cultures, while useful for the analysis of gene expression and eExG outgrowth, have been unable to properly show urethral masculinization^40,41^. The current slice culture system recapitulates in vivo urethral masculinization including fusion of the UPE, male-specific mesenchymal cell migration and androgen-inducible expression of several eExG masculinizing genes. To our knowledge, this is the first report that a slice culture method was applied successfully to analyze androgen-driven sexually dimorphic organogenesis. Currently, slice cultures have extensively been used to study brain-related biological processes^42^. It is possible that a similar slice culture method may be applied for the visualization of developmental processes of other organs.

In summary, actomyosin contractility plays an important role for androgen-driven urethral masculinization. Actomyosin contractility is necessary for the androgen-driven and sexually dimorphic condensation of the male mesenchyme. Additionally, actomyosin-regulated mesenchymal condensations contribute to urethral epithelium morphogenesis. These findings further highlight the importance of mesenchymal dynamics for epithelial morphogenesis as a mechanism for the regulation of sexually dimorphic organogenesis.

## Methods

### Animals

All animals were bred and maintained according to the regulations outlined by the Animal Research Committee of Wakayama Medical University and Kumamoto University, Japan. The embryos of pregnant mice were staged according to the day when a vaginal plug was observed, which was designated as E0.5. *Myh9*^*lox/lox*^ ^43^, *Myh10*^*lox/lox*^^44^, *Sall1*^*CreERT2*^^45^, *Shh*^*CreERT2*^^46^, and *R26R-EYFP*^47^ mice were utilized in this study. These mice were maintained in a C57BL/6J genetic background. E14.5-E18.5 male and female mouse embryos were analyzed. Deletion of the floxed sequence was performed through administration of tamoxifen (Sigma-Aldrich). Tamoxifen was dissolved in sesame oil and treated to pregnant mice (200 mg/kg body weight) through oral gavage at E9.5. ICR mice were utilized for the slice culture and for analysis of sexually dimorphic expression of MYH10 and p-MLC.

### eExG slice culture

Mouse eExG were dissected in PBS at E15.5 and embedded in 4% (wt/vol) low melting point agarose/1× PBS using Tissue-tek cryomolds. Gels were melted through microwave and subsequently cooled to 38 **°**C in water bath for one hour before embedding. After placing the tissue within the cryomolds, the set-up cassettes were placed on ice until the gel has solidified. The resulting gel block was then removed from the cryomold, trimmed and glued unto a tissue holder. Tissues were sliced in 1× PBS at 150 µm thickness using a 7000smz-vibratome (Campden Instruments). *Z*-axis deflection was adjusted minimally (less than 0.3 mm) to preserve tissue viability. Slices were placed on the Millicell Cell Culture Insert (EMD Millipore), which were subsequently placed on culture media (DMEM, 10% charcoal treated FBS, 1% penicillin-streptomycin) containing 10^-8^ M 5α-dihydrotestosterone (DHT) (Sigma-Aldrich) or equivalent volumes of the vehicle solution DMSO (Sigma-Aldrich). eExG slices were cultured within the air-liquid interface with one side of the tissue exposed to air and the other in contact with the media. Cultures were then incubated at 37 **°**C, 5% CO_2_. After 48 h, urethral masculinization scores were analyzed through bright field microscopy and histological analyses by hematoxylin and eosin (H&E) staining. Each eExG slice culture were assigned a qualitative Score from 1 (no visible fusion) to 4 (complete fusion) based on the extent by which the urethral epithelium has been removed resulting in mesenchymal confluence and urethral tubularization. Scoring was performed as follows: Score 1: no tubularization of the urethra nor removal of the UPE can be observed; Score 2: tubular urethra forms only in the surface regions of the slice tissue or less than ~20% of the tissue, unfused UPE can still be observed throughout most of the tissue; Score 3: tubular urethra develops in more than ~50% of the tissue, albeit the mesenchymal seam is not clearly formed (the mesenchymal seam is shown in Fig. 1a; red arrow); Score 4: tubular urethra develops throughout the entire tissue and the mesenchymal seam forms clearly (Supplementary Fig. 2). Score 0: no tubularization of the urethra nor removal of the UPE can be observed and slices show abnormal UPE morphologies that are not usually observed in wild type mouse eExG in vivo. Scores 3–4 were considered to indicate successful urethral masculinization while Scores 0–2 were assigned impaired masculinization. Whole images of eExG slice cultures were taken with an Olympus IX71 microscope and analyzed with NIS Elements BR 64 bit 3.22.00 software (Build 710, Laboratory Imaging, Nikon). Live imaging of urethral masculinization was performed using a Confocal Scanner Box, CellVoyager^TM^CV1000 microscope and resulting images were analyzed with Cell Voyager^TM^ CV1000 Software (Yokogawa Electric Corporation). Image processing and analysis of individual cell movements were performed using Imaris software (Imaris Track, Imaris Measurement Pro, and Imaris XT modules, after ver. 7) (Bitplane)^48^.

### Actomyosin inhibition

Blebbistatin (Sigma-Aldrich) was dissolved in DMSO to a stock concentration of 100 mM. Stock blebbistatin was then added to culture media to a final concentration of 100 µM. Male eExG slice cultures were treated with either 100 µM blebbistatin (with 10^−8^ M DHT) or with equivalent volumes of DMSO (with 10^−8^ M DHT). Slice cultures were subsequently scored for urethral masculinization after 48 h of incubation.

### Histology and immunostaining

Tissue samples were fixed in 4% (wt/vol) PFA overnight, dehydrated in serial methanol washes and embedded in paraffin. Paraffin blocks containing the tissue samples were sectioned serially (6 µm thick), placed on slides, deparaffinized and rehydrated. Antigen retrieval was performed with either citrate buffer, autoclaved at 121 **°**C, 1 min or Histo VT One (Naclai Tesque, Inc.), autoclaved at 105 **°**C, 15 min. Tissues were incubated with primary antibodies, MAFB (rabbit, 1/1500, IHC-00351, Bethyl Laboratories), AR (rabbit, 1/100, N-20SC-816, Santa Cruz Biotechnology), β-CATENIN (mouse, 1/100, 610153, BD Transduction Laboratory), MYH10 (rabbit, 1/500, PRB-445P, BioLegend), MYH9 (rabbit, 1/1000, ab24762, Abcam) p-MLC S20 (rabbit, 1/500, ab2480, Abcam), N-CADHERIN (mouse, 1/100, 610920, BD Transduction Laboratory), E-CADHERIN (mouse, 1/100, 610182, BD Transduction Laboratory), GFP (rabbit, 1/500, ab6556, Abcam), SALL1 (mouse, 1/200, PP-K9814-00, Perseus Proteomics). For immunofluorescence staining, primary antibodies were detected with Alexa Fluor 546-conjugated or 488-conjugated IgG (Molecular Probes, Oregon) and counterstained with Hoechst 33342 (Sigma-Aldrich). For primary antibodies raised in mouse, staining was performed using M.O.M. Immunodetection kit (Vector Laboratories) according to manufacturer’s suggested protocols with some modifications. For immunohistochemical staining, amplification using Vectastain ABC kit (Vector Laboratories) and TSA Indirect kit (Perkin Elmer) was performed. Signal was visualized through incubation with diaminobenzidine solution (Wako Pure Chemical Industries Ltd.). Images were visualized using an Olympus BX51 Fluorescence microscope and analyzed with Cell Sens Standard software (ver. 1.6, Olympus). Confocal images were visualized using a Carl Zeiss LSM 700 laser scanning confocal microscope and analyzed with Zen 2012 SP1 black edition 64-bit software (ver. 8.1, Carl Zeiss).

### Data analyses

Associations between treatment condition and urethral masculinization scores were analyzed through cross-tabulation. Statistical significance was tested using Chi-Square test (two-tailed values of *p* < 0.05 were considered to be significant) with Graphpad software (https://www.graphpad.com/quickcalcs/contingency1/).

### Reporting summary

Further information on experimental design is available in the Nature Research Reporting Summary linked to this article.

## Supplementary information

Supplementary Information

Reporting Summary

Supplementary Movie 1

Supplementary Movie 2

Supplementary Movie 3

Supplementary Movie 4

Supplementary Movie 5

Supplementary Data 1

## Data Availability

All data generated or analyzed during this study are included in this published Article and its Supplementary files.
