# Supplementary material for: Mesenchymal actomyosin contractility is required for androgen-driven urethral masculinization in mice
Source: Commun Biol. 2019 Mar 8;2:95. doi: 10.1038/s42003-019-0336-3 (PMC6408527; doi:10.1038/s42003-019-0336-3)
Supplement: Supplementary file 1 — Supplementary Information [file 42003_2019_336_MOESM1_ESM.pdf]

Supplementary Fig. 1

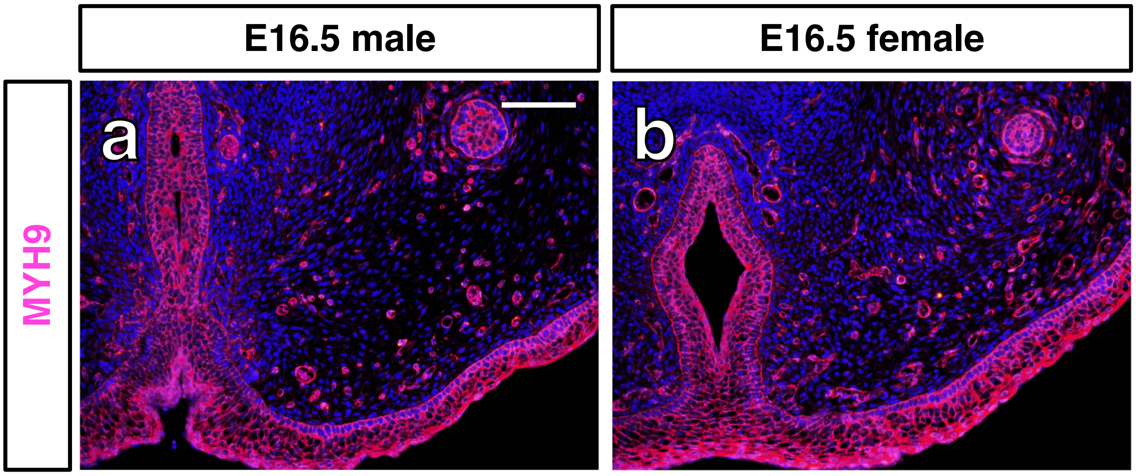

Supplementary Fig. 1. MYH9 expression in the (**a**) E16.5 male eExG and (**b**) E16.5 female eExG. MYH9 shows prominent expression in the epithelia of both male and female eExG. Scale bar in **a** = 100 μm.

Supplementary Fig. 2

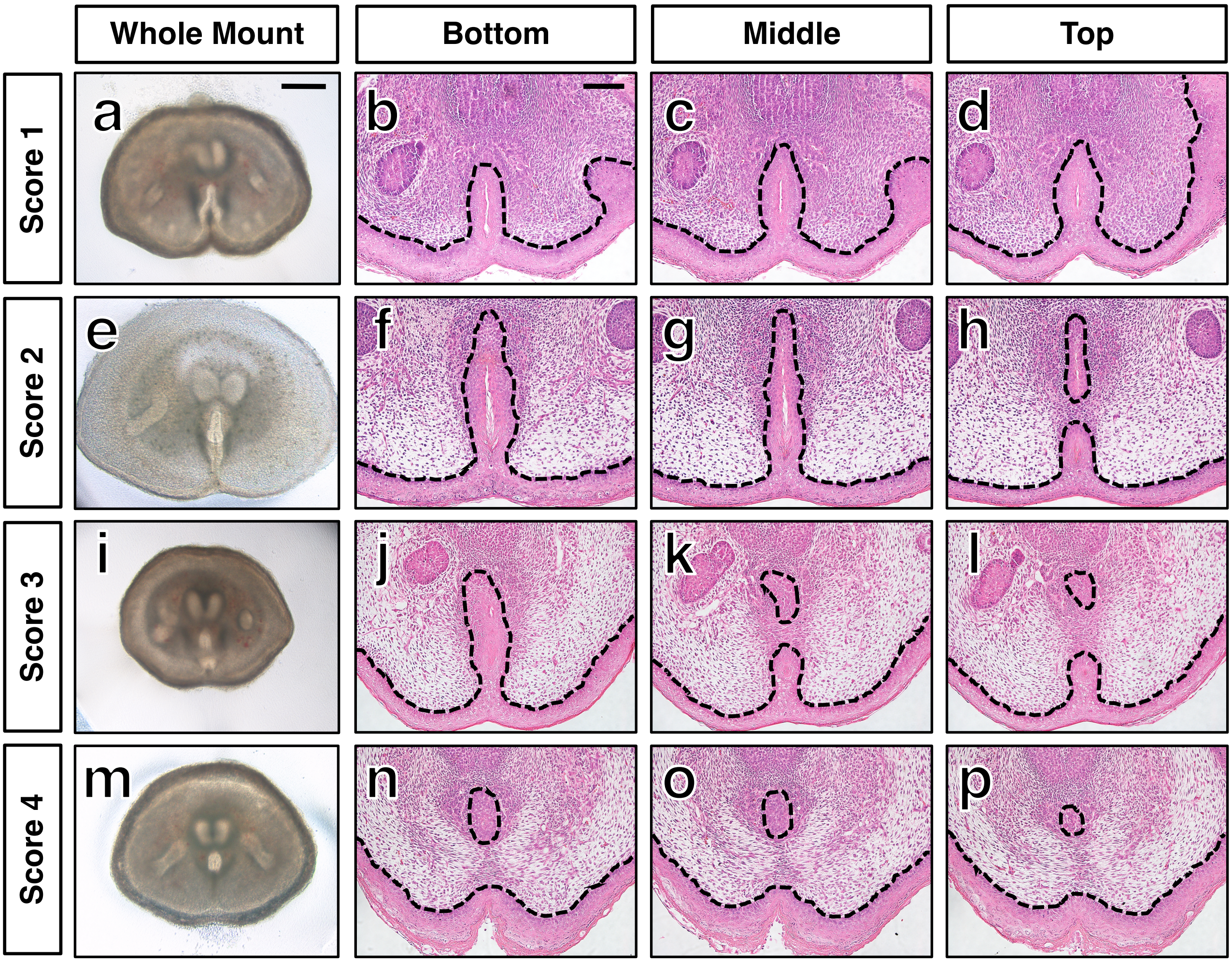

Supplementary Fig. 2. Histology of representative eExG slice cultures showing each Urethral Masculinization Score: (**a-d**) Score 1 eExG slice, (**e-h**) Score 2 eExG slice, (**i-l**) Score 3 eExG slice and (**m-p**) Score 4 eExG slice. (**a, e, i, m**) Whole mount and H&E staining showing urethral histology from the (**b, f, j, n**) bottom, (**c, g, k, o**) middle and (**d, h, l, p**) top region of the eExG slice tissue after 48 hours. Dashed lines; epithelial-mesenchymal border. Scale bar in **a** = 250 μm, scale bar in **b** = 100 μm.

Supplementary Fig. 3

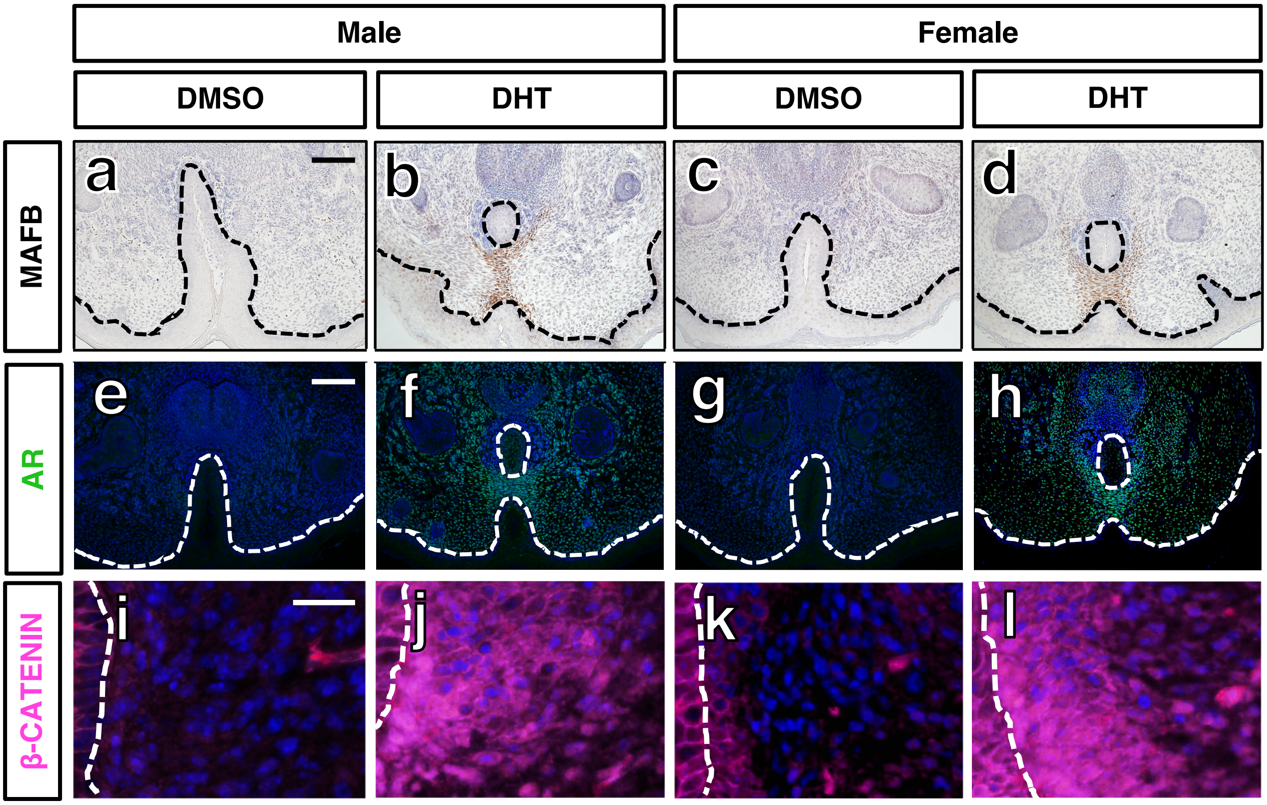

Supplementary Fig. 3. Treatment with DHT induces expression of masculinizing genes in eExG slices. (**a-d**) Expression of MAFB in (**a**) DMSO-treated male eExG slice (n = 3 eExG slices), (**b**) DHT-treated male eExG slice (n = 4 eExG slices), (**c**) DMSO-treated female eExG slice (n = 3 eExG slices) and (**d**) DHT-treated female eExG slice (n = 3 eExG slices) after 48 hours culture. (**e-h**) Expression of AR in (**e**) DMSO-treated male eExG slice (n = 4 eExG slices), (**f**) DHT-treated male eExG slice (n = 4 eExG slices), (**g**) DMSO-treated female eExG slice (n = 4 eExG slices) and (**h**) DHT-treated female eExG slice (n = 4 eExG slices) after 48 hours culture. (**i-l**) Expression of β-CATENIN in (**i**) DMSO-treated male eExG slice (n = 3 eExG slices), (**j**) DHT-treated male eExG slice (n = 5 eExG slices), (**k**) DMSO-treated female eExG slice (n = 5 eExG slices) and (**l**) DHT-treated female eExG slice (n = 5 eExG slices) after 48 hours culture. Dashed lines; epithelial mesenchymal border. Scale bars in **a, e** = 100 μm, scale bar in **i** = 20 μm.

Supplementary Fig. 4

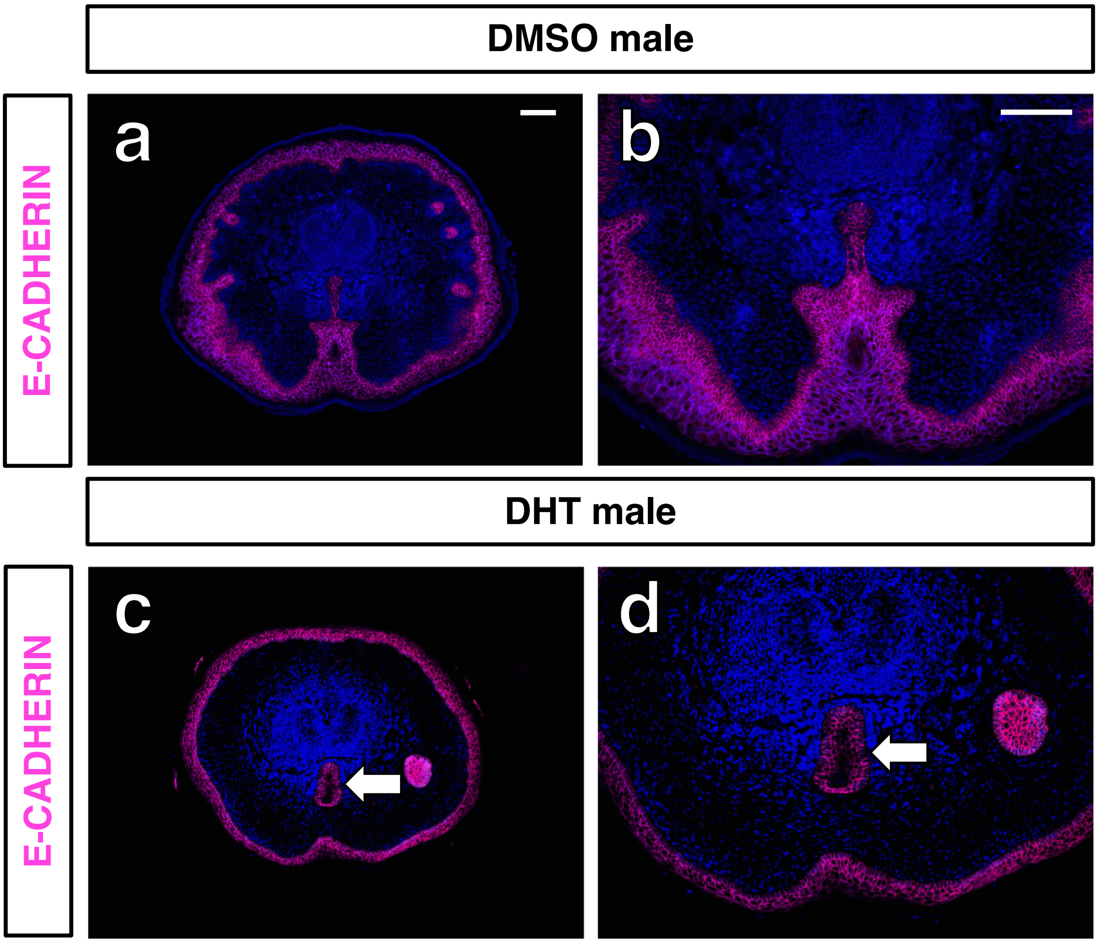

Supplementary Fig. 4. E-CADHERIN is expressed in the epithelium of (**a**, **b**) DMSO-treated and (**c**, **d**) DHT-treated male eExG slices after 48 hours (n = 3 eExG slices). E-CADHERIN expression indicates retention of epithelial integrity regardless of treatment with DHT. Tubular urethra (white arrows) forms in the eExG slice during culture as a result of DHT treatment. Scale bars = 100 μm.

Supplementary Fig. 5

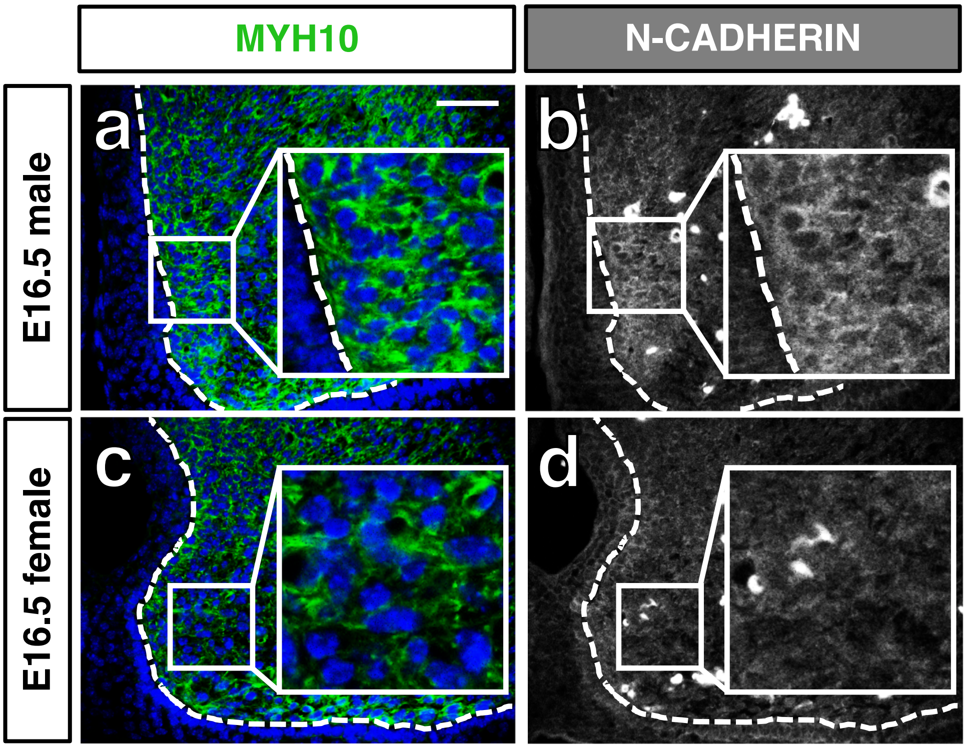

Supplementary Fig. 5. Sexually dimorphic expression of MYH10 and N-CADHERIN in the eExG bilateral mesenchyme. (**a**, **b**) E16.5 male eExG showing expression of (**a**) MYH10 and (**b**) N-CADHERIN. (**c**, **d**) E16.5 female eExG showing lower expression of (**c**) MYH10 and (**d**) N-CADHERIN. Square inserts; magnified view of bilateral mesenchyme. Dashed lines; epithelial mesenchymal border. Scale bar = 50 µm.

Supplementary Fig. 6

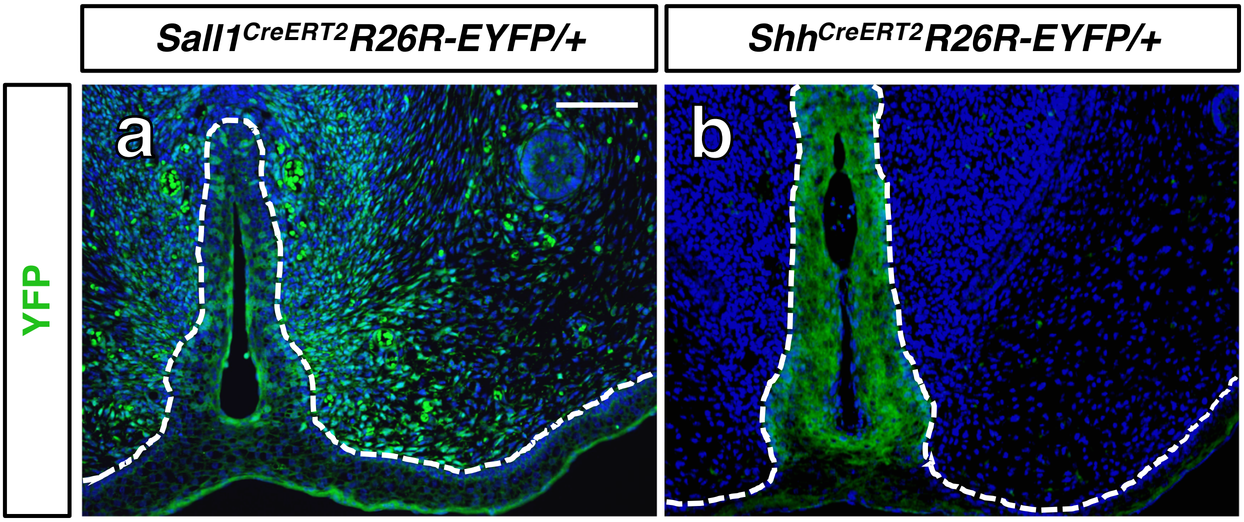

Supplementary Fig. 6. Immunostaining for YFP showing *Cre* recombinase expression in male offspring of *Sall1 CreERT2* or *Shh CreERT2* driver mice mated with *R26R-EYFP* mice. YFP expression in (**a**) E16.5 male *Sall1 CreERT2 R26R-EYFP/+* eExG (n = 2 eExG) and (**b**) E17.5 male *Shh CreERT2 R26R-EYFP/+* eExG (n = 3 eExG). Tamoxifen (200 mg/kg body weight) was administered to pregnant mice at E9.5. Dashed lines; epithelial mesenchymal border. Scale bar = 100 µm.

Supplementary Fig. 7

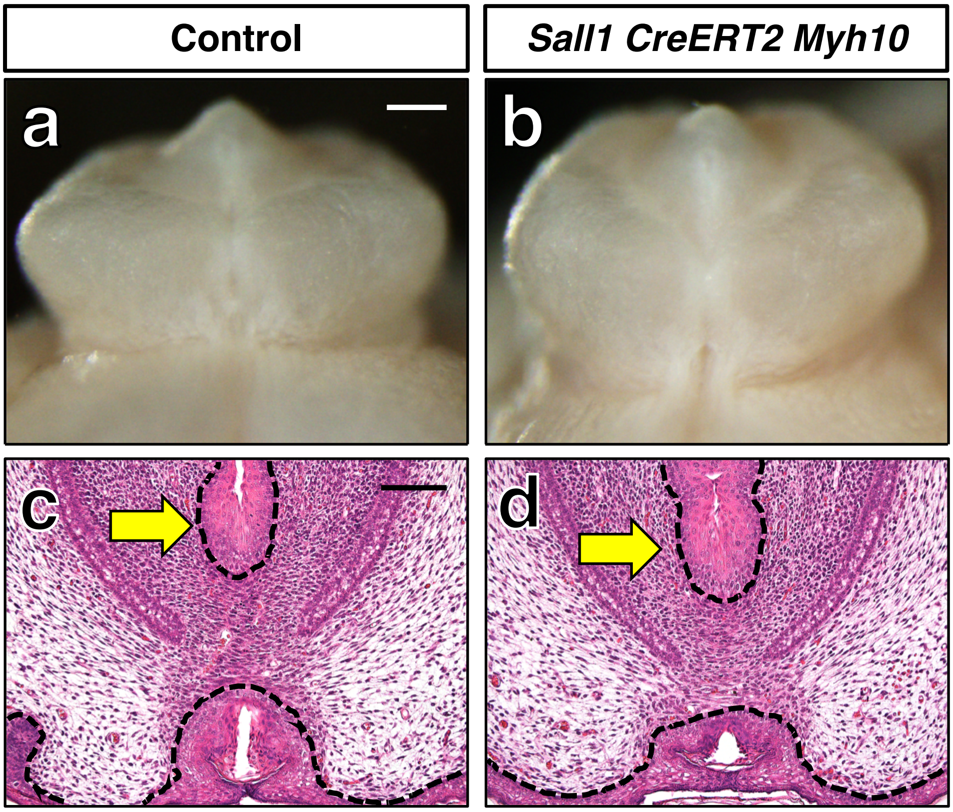

Supplementary Fig. 7. Mesenchymal deletion of only *Myh10* did not induce prominent defects in urethral tube formation. (**a, b**) Whole images and (**c-d**) H&E staining of E17.5 male eExG. (**a, c**) Control eExG (*Myh10$^{lox/lox}$*) and (**b, c**) *Sall1 CreERT2 Myh10* eExG (n = 15 eExG). Tubular urethra (yellow arrows) forms normally in both genotypes. Tamoxifen (200 mg/kg body weight) was administered to pregnant mice at E9.5. Dashed lines; epithelial mesenchymal border. Scale bar in **a** = 250 µm, scale bar in **c** = 100 µm.

Supplementary Fig. 8

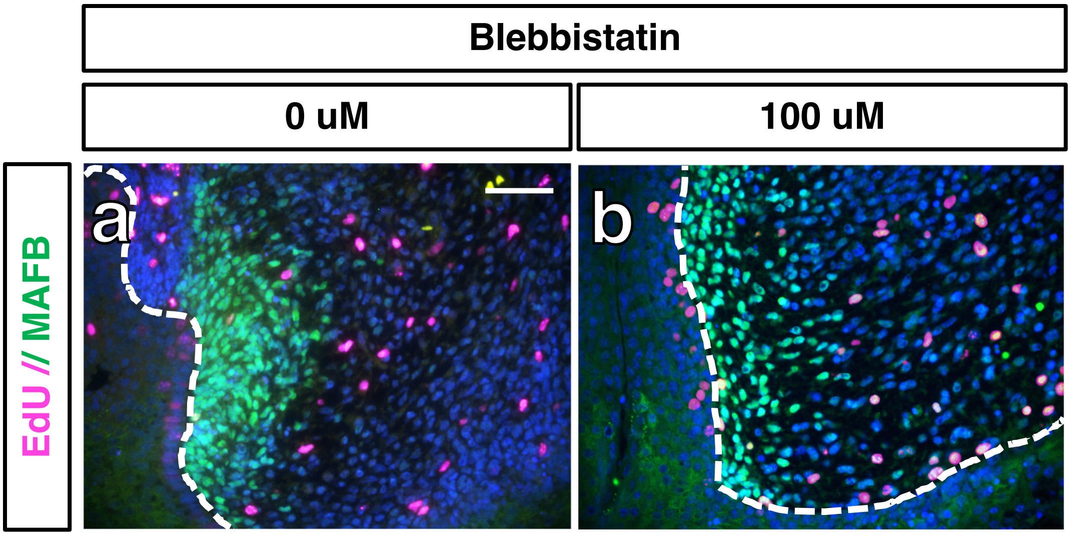

Supplementary Fig. 8. Analysis of cell proliferation by EdU incorporation in blebbistatin-treated male eExG slice culture. Blebbistatin treatment did not cause prominent changes in EdU incorporation. (**a**) Control male eExG slice (n = 7 eExG slices) and (**b**) blebbistatin-treated male eExG slice (n = 7 eExG slices) after 48 hours culture with DHT. Dashed lines; epithelial mesenchymal border. Scale bar in **a** = 50 μm.

Supplementary Table 1. Summary of genes involved in defects in external genitalia development.

Gene	Knockout phenotype	Reference
Ar	Feminization of the external genitalia	1
Fgf10	Defects of urethra formation<br>- ventrally open embryonic urethra<br>- prepuce fusion defects	2
Shh	Agenesis of the external genitalia<br>Defective cloaca formation with anorectal malformations	3-5
β-catenin	Reduced outgrowth of external genitalia<br>Defects of urethral formation<br>- ventrally open embryonic urethra (proximal)<br>- abnormality of distal urethra formation	6
Mafb	Defects of urethral formation<br>- ventrally open embryonic male urethra (hypospadias-like phenotype)	7
Hoxa13	Defects of urethral formation<br>- ventrally open embryonic urethra	8
Fkbp52	Defects in the elevation of preputial swelling<br>Defects of urethral formation<br>- ventrally open embryonic urethra	9
Fgfr	Hypoplastic prepuce<br>Reduced outgrowth of external genitalia<br>Defects of urethral formation<br>- ventrally open embryonic urethra	10, 11
Dlx5/6	Defects of urethral formation<br>- ventrally open embryonic urethra	12

Supplementary Methods

Cell proliferation

Analysis of cell proliferation was performed with the Click-iT EdU Imaging kit (C10338; Invitrogen) according to manufacturer's instructions. Slice cultures were incubated with 10 μM EdU in culture media for two hours. Tissue samples were fixed in 4% (wt/vol) PFA overnight, dehydrated in serial methanol washes and embedded in paraffin. Paraffin blocks containing the tissue samples were sectioned serially (6 μm thick), placed on slides, deparaffinized and rehydrated. Antigen retrieval was performed with citrate buffer, autoclaved at 121°C, 1 min. Tissues were then incubated with the primary antibody, MAFB (rabbit, 1/1500, IHC-00351, Bethyl Laboratories) which was detected with Alexa Fluor 488-conjugated IgG (Molecular Probes, Oregon). Prepared slides were subsequently incubated with the Click-iT Reaction Cocktail (C10338; Invitrogen) for 30 minutes to detect EdU incorporation and counterstained with Hoechst 33342 (Sigma-Aldrich). Images were visualized using an Olympus BX51 Fluorescence microscope and analyzed with Cell Sens Standard software (ver. 1.6, Olympus).

References for Supplementary Table 1

1 Zheng, Z., Armfield, B. A. & Cohn, M. J. Timing of androgen receptor disruption and estrogen exposure underlies a spectrum of congenital penile anomalies. *Proc Natl Acad Sci U S A* **112**, E7194-7203, doi:10.1073/pnas.1515981112 (2015).

2 Haraguchi, R. *et al.* Molecular analysis of external genitalia formation: the role of fibroblast growth factor (Fgf) genes during genital tubercle formation. *Development* **127**, 2471-2479 (2000).

3 Haraguchi, R. *et al.* Unique functions of Sonic hedgehog signaling during external genitalia development. *Development* **128**, 4241-4250 (2001).

4 Seifert, A. W., Bouldin, C. M., Choi, K. S., Harfe, B. D. & Cohn, M. J. Multiphasic and tissue-specific roles of sonic hedgehog in cloacal septation and external genitalia development. *Development* **136**, 3949-3957, doi:10.1242/dev.042291 (2009).

5 Miyagawa, S. *et al.* The role of sonic hedgehog-Gli2 pathway in the masculinization of external genitalia. *Endocrinology* **152**, 2894-2903, doi:10.1210/en.2011-0263 (2011).

6 Lin, C., Yin, Y., Long, F. & Ma, L. Tissue-specific requirements of beta-catenin in external genitalia development. *Development* **135**, 2815-2825, doi:10.1242/dev.020586 (2008).

7 Suzuki, K. *et al.* Sexually dimorphic expression of Mafb regulates masculinization of the embryonic urethral formation. *Proc Natl Acad Sci U S A* **111**, 16407-16412, doi:10.1073/pnas.1413273111 (2014).

8 Morgan, E. A., Nguyen, S. B., Scott, V. & Stadler, H. S. Loss of Bmp7 and Fgf8 signaling in Hoxa13-mutant mice causes hypospadia. *Development* **130**, 3095-3109 (2003).

9 Chen, H. *et al.* Fkbp52 regulates androgen receptor transactivation activity and male urethra morphogenesis. *J Biol Chem* **285**, 27776-27784, doi:10.1074/jbc.M110.156091 (2010).

10 Gredler, M. L., Seifert, A. W. & Cohn, M. J. Tissue-specific roles of Fgfr2 in development of the external genitalia. *Development* **142**, 2203-2212, doi:10.1242/dev.119891 (2015).

11 Harada, M. *et al.* Tissue-specific roles of FGF signaling in external genitalia development. *Dev Dyn* **244**, 759-773, doi:10.1002/dvdy.24277 (2015).

12 Suzuki, K. *et al.* Abnormal urethra formation in mouse models of split-hand/split-foot malformation type 1 and type 4. *Eur J Hum Genet* **16**, 36-44, doi:10.1038/sj.ejhg.5201925 (2008).
