# Supplementary material for: Mesenchymal actomyosin contractility is required for androgen-driven urethral masculinization in mice
Source: Commun Biol. 2019 Mar 8;2:95. doi: 10.1038/s42003-019-0336-3 (PMC6408527; doi:10.1038/s42003-019-0336-3)
Supplement: Supplementary file 2 — Reporting Summary [file 42003_2019_336_MOESM2_ESM.pdf]

natureresearch

Corresponding author(s): Gen Yamada

Reporting Summary

Nature Research wishes to improve the reproducibility of the work that we publish. This form provides structure for consistency and transparency in reporting. For further information on Nature Research policies, see Authors & Referees and the Editorial Policy Checklist.

Statistical parameters

When statistical analyses are reported, confirm that the following items are present in the relevant location (e.g. figure legend, table legend, main text, or Methods section).

n/a	Confirmed	
☐	☒	The exact sample size (*n*) for each experimental group/condition, given as a discrete number and unit of measurement
☐	☒	An indication of whether measurements were taken from distinct samples or whether the same sample was measured repeatedly
☐	☒	The statistical test(s) used AND whether they are one- or two-sided<br>*Only common tests should be described solely by name; describe more complex techniques in the Methods section.*
☒	☐	A description of all covariates tested
☒	☐	A description of any assumptions or corrections, such as tests of normality and adjustment for multiple comparisons
☒	☐	A full description of the statistics including central tendency (e.g. means) or other basic estimates (e.g. regression coefficient) AND variation (e.g. standard deviation) or associated estimates of uncertainty (e.g. confidence intervals)
☐	☒	For null hypothesis testing, the test statistic (e.g. *F*, *t*, *r*) with confidence intervals, effect sizes, degrees of freedom and *P* value noted<br>*Give P values as exact values whenever suitable.*
☒	☐	For Bayesian analysis, information on the choice of priors and Markov chain Monte Carlo settings
☒	☐	For hierarchical and complex designs, identification of the appropriate level for tests and full reporting of outcomes
☒	☐	Estimates of effect sizes (e.g. Cohen's *d*, Pearson's *r*), indicating how they were calculated
☒	☐	Clearly defined error bars<br>*State explicitly what error bars represent (e.g. SD, SE, CI)*

Our web collection on statistics for biologists may be useful.

Software and code

Policy information about availability of computer code

Data collection	For collection of immunofluorescence and immunohistochemistry images:<br>Cell Sens Standard 1.6<br>Olympus Corporation<br><br>For collection of confocal immunofluorescence images:<br>Carl Zeiss Zen 2012 SP1 (black edition) (64 bit)<br>Release version 8.1<br><br>For collection of whole images of embryonic External Genitalia (eExG):<br>DPController 1.2.1.108<br>Olympus Optical CO, LTD.<br><br>For collection of whole images of eExG slice cultures:<br>NIS Elements BR 64 bit 3.22.00 (Build 710)<br>Laboratory Imaging, Nikon<br><br>For collection of live imaging movies:<br>Cell VoyagerTM CV1000 Software<br>Yokogawa Electric Corporation

Data analysis	For analysis of immunofluorescence and immunohistochemistry images:<br>Cell Sens Standard 1.6<br>Olympus Corporation<br><br>For analysis of confocal immunofluorescence images:<br>Carl Zeiss Zen 2012 SP1 (black edition) (64 bit)<br>Release version 8.1<br><br>For analysis of live imaging movies (cell tracking)<br>Imaris software (Imaris Track, Imaris Measurement Pro, and Imaris XT modules, after ver. 7) (Bitplane)

For manuscripts utilizing custom algorithms or software that are central to the research but not yet described in published literature, software must be made available to editors/reviewers upon request. We strongly encourage code deposition in a community repository (e.g. GitHub). See the Nature Research guidelines for submitting code & software for further information.

Data

Policy information about availability of data

All manuscripts must include a data availability statement. This statement should provide the following information, where applicable:

- Accession codes, unique identifiers, or web links for publicly available datasets
- A list of figures that have associated raw data
- A description of any restrictions on data availability

All data generated or analysed during this study are included in this published article (and its Supplementary Information files).

Field-specific reporting

Please select the best fit for your research. If you are not sure, read the appropriate sections before making your selection.

☒ Life sciences ☐ Behavioural & social sciences ☐ Ecological, evolutionary & environmental sciences

For a reference copy of the document with all sections, see nature.com/authors/policies/ReportingSummary-flat.pdf

Life sciences study design

All studies must disclose on these points even when the disclosure is negative.

Sample size	For analysis of sexually dimorphic gene expression through immunofluorescence / immunohistochemistry, we analyzed at least around 3 pairs of male and female embryonic external genitalia (eExG) and a similar number of androgen-treated and untreated eExG slice cultures. This sample size is widely accepted as sufficient to confirm gene expression patterns.<br><br>For analysis of urethral masculinization in response to hormone treatment or pharmacologic inhibitors, at least 15-30 individual eExG slices for each treatment condition were analyzed. This sample size is quite sufficient for the qualitative analysis of urethral masculinization.<br><br>For analysis of mutant phenotypes, at least 15-30 pairs of mutant and control eExG were analyzed. This sample size is quite sufficient for the qualitative analysis of urethral masculinization.
Data exclusions	No data was excluded for this study.
Replication	At least around 3 independent biologically distinct replicates were utilized for each experiment. More than three sections from each biological eExG replicate were analyzed for immunofluorescence or immunohistochemistry. All attempts at replication were successful.
Randomization	All samples were allocated to treatment groups through simple randomization.
Blinding	For the analysis of male and female / mutant and control eExG, it was not possible to blind the investigator to group allocation during data collection / analysis because of the distinct phenotypes of each sample.<br>For analysis of eExG masculinzation in response to hormone treatment and pharmacologic inhibition, an investigator blinded to the treatment conditions of the samples was asked to perform the data analysis based on our definition for urethral masculinization (Fig. S1).

Reporting for specific materials, systems and methods

Materials & experimental systems

n/a	Involved in the study
☒	☐ Unique biological materials
☐	☒ Antibodies
☒	☐ Eukaryotic cell lines
☒	☐ Palaeontology
☐	☒ Animals and other organisms
☒	☐ Human research participants

Methods

n/a	Involved in the study
☒	☐ ChIP-seq
☒	☐ Flow cytometry
☒	☐ MRI-based neuroimaging

Antibodies

Antibodies used

MAFB (rabbit, 1/1500, Catalog No: IHC-00351, Lot: 1, Bethyl Laboratories)
AR (rabbit, 1/100, Catalog No: N-20SC-816, Lot: H1913, Santa Cruz Biotechnology)
β-CATENIN (mouse, 1/100, Catalog No: 610153, Lot: 81374, BD Transduction Laboratory)
MYH9 (rabbit, 1/1000, Catalog No: ab24762, Abcam)
MYH10 (rabbit, 1/500, Catalog No: PRB-445P, Lot: D14JF02050, BioLegend)
p-MLC S20 (rabbit, 1/500, Catalog No: ab2480, Lot: GR270454-8, Abcam)
N-CADHERIN (mouse, 1/100, Catalog No: 610920, Lot: 6134680, BD Transduction Laboratory)
E-CADHERIN (mouse, 1/100, Catalog No: 610182, Lot: 2307882, BD Transduction Laboratory)
GFP (rabbit, 1/500, Catalog No: ab6556, Lot: GR292567-1, Abcam)
SALL1 (mouse, 1/200, Catalog No: PP-K9814-00, Lot: A-3, Perseus Proteomics)

Validation

Each primary antibody was titrated against male eExG at three different concentrations. Expression patterns were assessed according to existing literature. Optimal concentrations were then selected for successive experiments. Negative controls that were not incubated with the primary antibody were utilized in order to confirm specificity of the observed signal.

For the sexually dimorphic genes MAFB, AR, β-CATENIN and SALL1, validation of the antibody was performed by confirming expression in the male vs. female eExG.

For MYH10, validation was performed by confirming the reduced expression in the current mutant mouse model.

MAFB, AR, β-CATENIN, GFP primary antibodies have been reported previously by our laboratory. SALL1 primary antibody has been reported previously by our collaborator.

REFERENCES

MAFB and AR: Suzuki, K., Numata, T., Suzuki, H., Raga, D. D., Ipulan, L. A., Yokoyama, C., Matsushita, S., Hamada, M., Nakagata, N., Nishinakamura, R., et al. (2014). Sexually dimorphic expression of Mafb regulates masculinization of the embryonic urethral formation. Proc Natl Acad Sci U S A 111, 16407-16412.

β-CATENIN: Suzuki, H., Matsushita, S., Suzuki, K. and Yamada, G. (2017). 5α-Dihydrotestosterone negatively regulates cell proliferation of the periurethral ventral mesenchyme during urethral tube formation in the murine male genital tubercle. Andrology 5, 146-152.

GFP: Ipulan, L. A., Suzuki, K., Sakamoto, Y., Murashima, A., Imai, Y., Omori, A., Nakagata, N., Nishinakamura, R., Valasek, P. and Yamada, G. (2014). Nonmyocytic androgen receptor regulates the sexually dimorphic development of the embryonic bulbocavernosus muscle. Endocrinology 155, 2467-2479.

SALL1: Recuenco, M. C., Ohmori, T., Tanigawa, S., Taguchi, A., Fujimura, S., Conti, M. A., Wei, Q., Kiyonari, H., Abe, T., Adelstein, R. S., et al. (2015). Nonmuscle Myosin II Regulates the Morphogenesis of Metanephric Mesenchyme-Derived Immature Nephrons. J Am Soc Nephrol 26, 1081-1091.

Animals and other organisms

Policy information about studies involving animals; ARRIVE guidelines recommended for reporting animal research

Laboratory animals

3-6 months old C57BL/6J and ICR mice were utilized in the generation of E14.5, E15.5, E16.5 and E18.5 embryos.

Wild animals

The study did not involve wild animals.

Field-collected samples

The study did not involve samples collected from the field.
